# Brain organoid-on-chip system to study the effects of breast cancer derived exosomes on the neurodevelopment of brain

**DOI:** 10.1186/s13619-021-00102-7

**Published:** 2022-03-07

**Authors:** Kangli Cui, Wenwen Chen, Rongkai Cao, Yingying Xie, Peng Wang, Yunsong Wu, Yaqing Wang, Jianhua Qin

**Affiliations:** 1 Division of Biotechnology, Dalian Institute of Chemical Physics, Chinese Academy of Sciences, Dalian, China; 2 University of Chinese Academy of Sciences, Beijing, China; 3 Institute for Stem Cell and Regeneration, Chinese Academy of Sciences, Beijing, China; 4 CAS Center for Excellence in Brain Science and Intelligence Technology, Chinese Academy of Sciences, Shanghai, China

**Keywords:** Brain organoid, Human induced pluripotent stem cell, Breast cancer, Exosomes

## Abstract

Early human brain development can be affected by multiple prenatal factors that involve chemical exposures *in utero,* maternal health characteristics such as psychiatric disorders, and cancer. Breast cancer is one of the most common cancers worldwide arising pregnancy. However, it is not clear whether the breast cancer might influence the brain development of fetus. Exosomes secreted by breast cancer cells play a critical role in mediating intercellular communication and interplay between different organs. In this work, we engineered human induced pluripotent stem cells (hiPSCs)-derived brain organoids in an array of micropillar chip and probed the influences of breast cancer cell (MCF-7) derived-exosomes on the early neurodevelopment of brain. The formed brain organoids can recapitulate essential features of embryonic human brain at early stages, in terms of neurogenesis, forebrain regionalization, and cortical organization. Treatment with breast cancer cell derived-exosomes, brain organoids exhibited enhanced expression of stemness-related marker OCT4 and forebrain marker PAX6. RNA-seq analysis reflected several activated signaling pathways associated with breast cancer, medulloblastoma and neurogenesis in brain organoids induced by tumor-derived exosomes. These results suggested that breast cancer cell-derived exosomes might lead to the impaired neurodevelopment in the brain organoids and the carcinogenesis of brain organoids. It potentially implies the fetus of pregnant women with breast cancer has the risk of impaired neurodevelopmental disorder after birth.

**Supplementary Information:**

The online version contains supplementary material available at 10.1186/s13619-021-00102-7.

## Background

The development of human brain is extremely rapid during the period of fetus and the first year of life. The fetal brain are susceptible to a series of prenatal factors, such as maternal health characteristics during pregnancy (Pulli et al. 2019). Multiple maternal health issues, such as prenatal maternal depression, maternal obesity, and cancers, have a negative effect on the *in utero* brain development. Breast cancer, a heterogeneous disease, exhibited extensive genomic, transcriptomic and proteomic alterations (Groza et al. 2020). It is one of the malignant tumors arising in pregnancy with greater than 450, 000 deaths each year worldwide (Koboldt et al. 2012). Previous studies indicated that exosomes purified from breast cancer cells resulted in increased angiogenesis, invasion, and migration in cells (Groza et al. 2020).

Exosomes are extracellular vesicles containing constituents (lipids, proteins and miRNAs). They can be absorbed by distant cells, where they can influence the cell function and behavior. Exosomes can mediate intercellular communication in pathology (Hoshino et al. 2020) and be involved in the pathogenesis of cancer (Kalluri and LeBleu 2020), by providing a novel paracrine signaling mechanism during tumor progression (King et al. 2012). Exosomes promoted tumor progression by mediating communication between the tumor and surrounding stromal tissue (Webber et al. 2010). In addition, they activated the proliferative and angiogenic pathways (Qu et al. 2009; Skog et al. 2008), by bestowing immune suppression (Clayton et al. 2008; Liu et al. 2006), and initiation of pre-metastatic sites (Hood et al. 2011). However, it is not clear whether tumor-derived exosomes influence the neurodevelopment of brain.

**Fig. 1:**
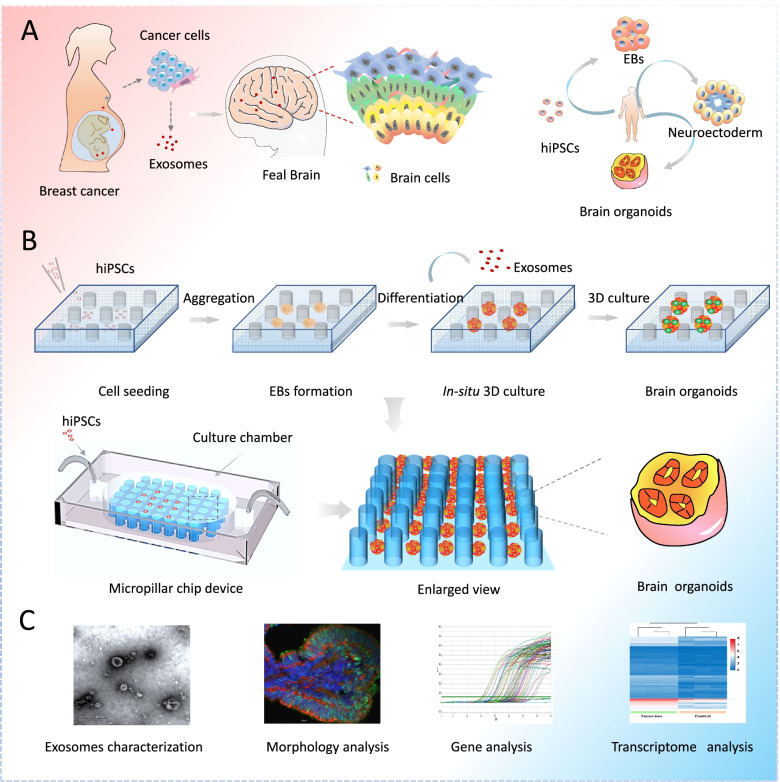
Illustrations of engineered human brain organoids for probing the effects of breast cancer-derived exosomes on neurodevelopment in brain at early stages. **A** Illustration of procedures of formation of brain organoids from self-assembly of hiPSCs *in vitro*. **B** Procedures of generation of hiPSCs-derived brain organoids on a micropillar chip device. **C **Methods used to exemplify the effect of tumor cell derived exosomes on the brain organoids

Brain organoids are self-assembled 3D clusters from human pluripotent stem cells (hPSCs) (Amin and Paşca 2018). They recapitulate the essential cell types and architectures of the embryonic human brain at early stages (Yin et al. 2018; Zhu et al. 2017; Wang et al. 2018). In this study, we established an engineered cortical organoid model from human induced pluripotent stem cells (hiPSCs) in a micropillar array chip and explored the effects of exosomes from breast cancer cell on the neurodevelopment of brain (Fig. 1 A, B).

Initially, the purified exosomes were identified by means of particle size analysis, transmission electron microscope (TEM) and western blot. The engineered brain organoids were identified in terms of neural differentiation, regionalization and cortical organization by immunofluorescence analysis. Subsequently, the effects of exosomes derived from breast cancer cell on the expression of stemness genes and forebrain differentiation in brain organoids were explored by quantitative real-time PCR (qRT-PCR) and immunofluorescence staining. Finally, the transcriptional responses of brain organoids to exosome exposure were characterized by RNA-sequencing (RNA-seq) analysis (Fig. 1C).

## Results 

### Extraction and identification of exosomes from breast cancer cell line

Prior to examine whether tumor-derived exosomes exert effect on the brain organoids, we initially extracted and purified the exosomes by ultracentrifugation according to previous article (György et al. 2011; Chen et al. 2019). Briefly, MCF-7 cells were cultured using medium supplemented with exosomes-free FBS, then were collected for centrifugation to remove cells, dead cells, cell debris, and microvesicles. Subsequently, the prepared samples were performed ultracentrifugation to purify the exosomes (Fig. S1A). To determine the particle size of exosomes, we conducted particle size analysis on the exosomes derived from MCF-7 using Zetasizer Nano. Nanoparticle tracking analysis demonstrated that particle size of the exosomes was around 100 nm (Fig. S1B). Furthermore, the biomarker proteins (CD9 and CD63) of exosomes were characterized by western blot, which demonstrated the marked expressions of CD9 and CD63 in MCF-7-derived exosomes (Fig. S1C). The morphology of exosomes was examined using TEM. It showed that the purified exosomes were elliptical or circular vesicles featured with cup- and disc-shaped structures (Fig. S1D). These results revealed that the purified exosomes from MCF-7 exhibited typical features of exosomes in terms of morphology, size distribution and exosome biomarkers.

### Characterizations of brain organoids derived from hiPSCs on chip

**Fig. 2:**
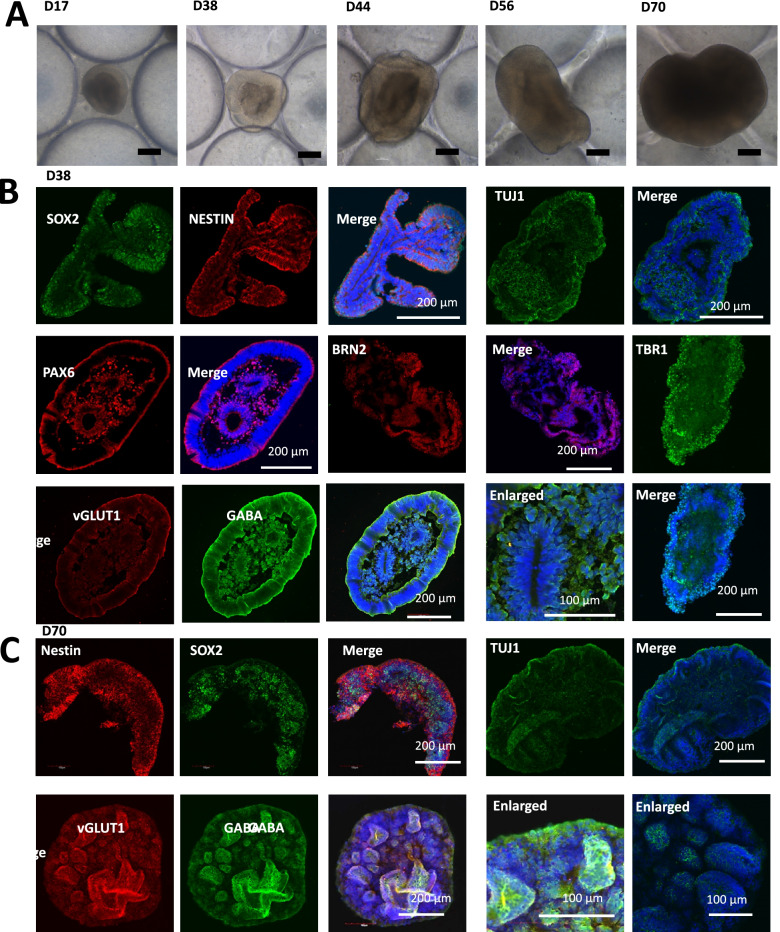
Characterization of brain organoids derived from hiPSC on the micropillar chip. **A** Bright-field images of developing brain organoids at different days. Scale bar: 200 μm. **B, C** Immunohistochemical analysis were conducted to detect the NESTIN^+^/SOX2^+^ neural progenitor cells, TUJ1^+ ^differentiated neurons, PAX6^+^ forebrain, BRN2^+^ and TBR1^+^ cortical layer, vGLUT1^+^ excitatory neurons and GABA^+^ inhibitory neurons in day-38 and day-70 brain organoids. Scale bars indicated in the image

Generally, the brain organoids were generated by the self-aggregation of hiPSCs that included the formation of embryoid bodies (EBs), neuroectoderm differentiation, and cortical organization. According to previous approach (Cui et al. 2020a), we modified and leveraged a simple micropillar array chip to engineer brain organoids from hiPSCs in a controlled manner, which allowed to EBs formation and *in situ* organization of brain organoids (Fig. 2A). To characterize the detailed features of neurogenesis during the period of brain organoids development, we investigated the efficiency of the early neuron differentiation. The neural progenitor cells (NPCs) markers (SOX2 and NESTIN) and neuron marker (TUJ1) were examined in brain organoids at distinct developmental stages by immunostaining analysis. As expected, high proportions of SOX2^+^, NESTIN^+^ and TUJ1^+^ cells were expressed in brain organoids at day 38, even in the day 70 organoids after prolonged development (Fig. 2B-C).

Furthermore, the expression of forebrain specific marker PAX6 at day 38 of organoid differentiation was remarkably upregulated, indicating the distribution of forebrain region (Fig. 2B). We next analyzed the characteristics of cortical organization, including the expression of deep layer neuron marker TBR1 and layer 3 neuron marker BRN2 via immunofluorescence staining. The brain organoids at days 38 showed high expressions of TBR1 and BRN2, implying the organization of the initial cortical structure containing deep neural layers (Fig. 2B). Additionally, the neuron subtypes inclusion of inhibitory neuron (GABA^+^) and excitatory neuron (vGLUT1^+^) were observed to expressed in brain organoids at day 38 and 70 by immunohistochemical analysis (Fig. 2B-C). These results suggest the efficient neural induction, forebrain regionalization and cortical structure formation in the formed organoids, which recapitulate the essential features of cell types in the human embryonic brain.

### Enhanced stemness of brain organoids under exosomes exposure

**Fig. 3:**
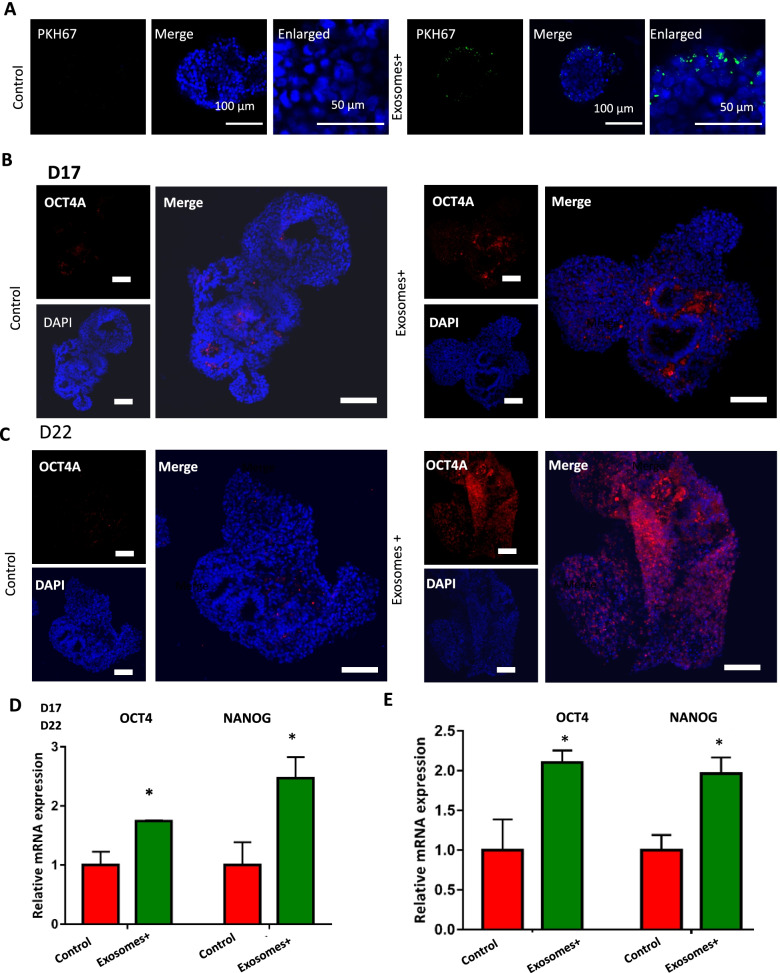
The impact of exosomes derived from breast cancer cell on the stemness of brain organoids. **A** Immunostaining images of cell membrane dyes PKH67 which used to mark exosomes in the developing brain organoids with and without exosome exposure. Scale bars:100 μm in the merge images and 50 μm in the enlarged images. **B, C** The Immunohistochemistry images of stemness marker: OCT4 in brain organoids with and without exosome exposure at day 17 and day 22. Scale bars: 100 μm. **D, E** The mRNA expression of stemness markers OCT4 and NANOG identified by qRT-PCR in brain organoids. Data are mean ± SEM. Student’s *t*-test, *P < 0.05

To explore the effects of tumor-derived exosomes on neurogenesis, the developing 3D clusters were exposed to exosomes that were labeled with PKH67 dye. PKH67 can emit green fluorescence and integrate into lipid bilayer membranes. After incubation with the dye for 24 h, labeled exosomes were markedly observed in perinuclear regions of cells by immunofluorescence staining, implying that brain organoids can take up the exosomes (Fig. 3A).

It is noted that, stemness markers (e.g., OCT4) of stem cell were found to be upregulated in tumor stem cells. But, during the differentiation of hiPSCs towards brain organoids, the stemness markers of stem cell in brain organoids tend to express seldom. We therefore investigated the effects of exosomes on the pluripotency of brain organoids during the developmental process. The day-12 brain organoids were treated without or with 50 μg/mL exosomes for 5 days and 10 days, respectively. Then, we detected the expression of stemness marker OCT4 of stem cells in the control and exosomes groups by immunofluorescence assay and qRT-PCR. The brain organoids showed higher populations of OCT4^+^ cells with exosomes exposure for 5 days in comparison to the control group by immunostaining analysis (Fig. 3B).

Consistently, qRT-PCR data demonstrated the significantly increased expression of stemness associated genes (NANOG and OCT4) of stem cell in organoids (Fig. 3D). With prolonged exosomes treatment for 10 days, the brain organoids exhibited similar response to exosomes exposure identified with higher expression of OCT4 compared to the control groups (Fig. 3C and E). These data suggested that tumor-derived exosomes incurred the increased stemness in the brain organoids characterized by the upregulated expression of OCT4. It was consistent with the previous study demonstrated mouse iPSCs exhibited features of cancer stem-like cells transformed by tumor-derived extracellular vesicles (Yan et al. 2014). This implies the capability of breast derived exosomes to lead to the cancer-like features in brain organoids. Further work is also required to verify these finding with *in vivo* models.

### Changes of forebrain differentiation in brain organoids

**Fig. 4:**
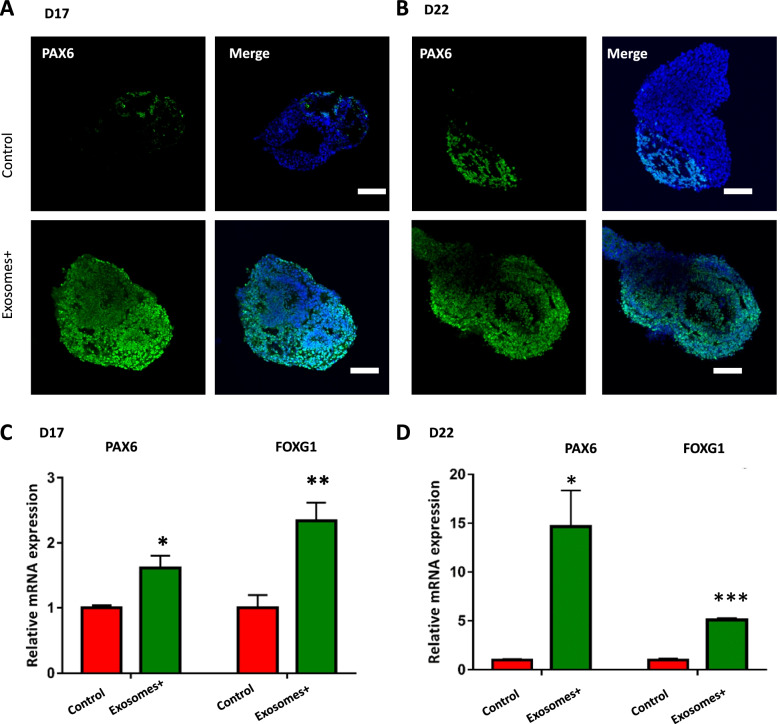
The effect of exosomes on the differentiation of forebrain region in brain organoids. **A**, **B** Immunohistochemistry image depicting the expression of forebrain related marker PAX6 in brain organoids under exosome exposure at days 17 and 22. Scale bars: 100 μm. **C**, **D** Transcripts for forebrain specific markers FOXG1 and PAX6 were examined by qRT-PCR in brain organoids under exosome exposure at day 17 and day 22. Data are mean ± SEM. Student’s *t*-test, *P < 0.05, **P < 0.01, ***P < 0.001

As brain development proceeded, we further investigated the effects of tumor-derived exosomes on the differentiation of forebrain region in brain organoids. The gene and protein expressions of the typical forebrain marker PAX6 were examined in brain organoids with or without exosomes exposure for 5 and 10 days. Immunohistochemical analysis demonstrated the significantly increased expression of PAX6 in exosomes-treated organoids compared to the control group (Fig. 4A-B). Consistently, the forebrain markers (PAX6 and FOXG1) were remarkably upregulated in organoids with exosomes exposure by qRT-PCR (Fig. 4C-D). These data implied that breast tumor-derived exosomes possibly contributed to the impairment of forebrain development. Previous study reported that PAX6 has oncogenic features and facilitates tumor progression (Jin et al. 2020) and FOXG1 has been reported to be elevated in the glioma (Chen et al. 2018) and dysfunction in the medulloblastoma (Adesina et al. 2007). It might imply tumor-derived exosomes conferred oncogenic features in brain organoids. This could be further validated with in vivo models.

### Transcriptome analysis of exosomes-treated brain organoids

**Fig. 5:**
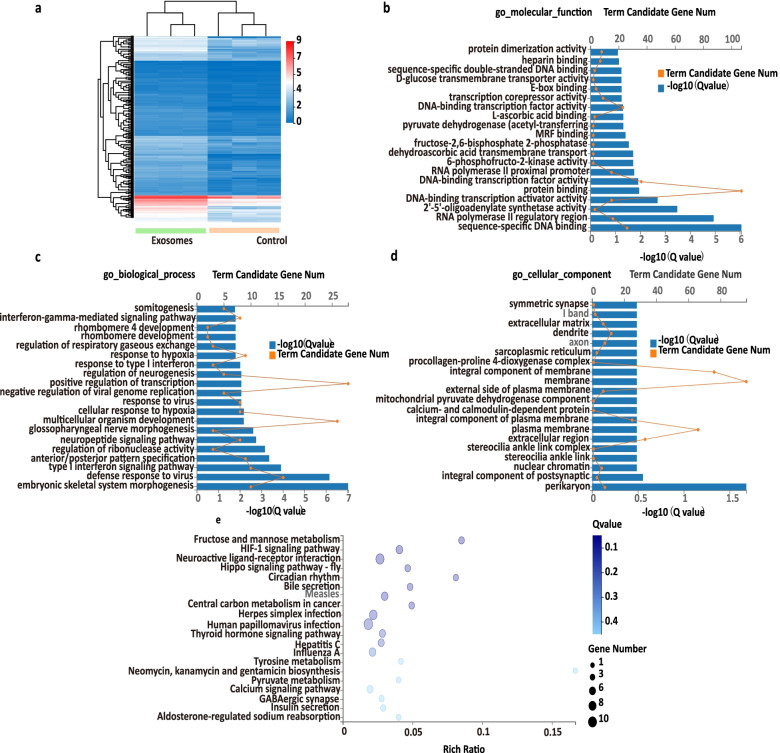
Transcriptome profiling of brain organoids exposed to breast cancer cell-derived exosomes. **A** Hierarchical clustering of unregulated DEGs in brain organoids treated with exosomes (the exosomes group) compared with the organoids without exosomes treatment (the control group). **B** Go enrichment of upregulated DEGs associated with molecular function in exosomes groups. **C** Go enrichment of upregulated DEGs associated with biological process in exosomes groups. **D** Go enrichment of upregulated DEGs related to cellular component in exosomes groups. **E** KEGG pathways of upregulated DEG in brain organoids exposed to exosomes. The x-axis and y-axis represent the rich ratio and the KEGG terms, respectively. The color and size of the circle indicates adjusted *P*-value and gene number, respectively

To fully understand the effect of tumor-derived exosomes on the brain organoids at global gene level, we performed RNA sequencing (RNA-seq). A total of 288 upregulated genes were identified in exosomes group based on the fold change was≥3 in mRNA expression (Fig. 5 A). Go enrichment of upregulated 288 differentially expressed genes (DEGs) were conducted based on cellular component, molecular function, and biological process. It revealed that exosomes exposure resulted in upregulated genes associated with molecular function like binding (Fig. 5B). Moreover, exosomes exposure enabled the unregulated genes related with biological process, such as response to type I interferon and regulation of neurogenesis (Fig. 5C). Furthermore, exosomes induced genes upregulated involved in cellular component, like synapse (Fig. 5D). KEGG (Kyoto Encyclopedia of Genes and Genomes) analysis demonstrated the enriched top pathways were associated with neuroactive ligand–receptor interactions, calcium signaling and metabolism (Fig. 5E).

Besides neuron signaling pathway, KEGG analysis of DEGs revealed enrichment of a multiple of cancer-related pathways, including pancreatic cancer, and breast cancer *etc* in organoids treated with exosomes (Fig. S2A). To identify the oncogenic signatures in brain organoids under exosomes exposure, MSigDB C6 were performed. It demonstrated that DEGs in brain organoids under exosomes exposure were enriched in breast cancer, medulloblastoma, and colon cancer pathway (Fig. S2B). The microRNA plays essential role in the progression of cancer. We therefore analyzed the target microRNA of DEGs in brain organoids under exosomes exposure using MSigDB C3. The data displayed that DEGs could target the motif of microRNA, including MIR511, MIR137, MIR218 *etc* (Fig. S2C), which has been reported to be essential role in the progression of cancer.

Additionally, we identified the expression of upregulated DEGs NPY4R, OAS1, HOXB1, and HOXC4 in brain organoids under exosomes exposure (Fig. S3). The expression pattern of NPY4R, OAS1, HOXB1, and HOXC4 is in line with the expression pattern of these genes by RNA-seq. The result further validated our transcriptomic findings. NPY4R is a gene for the appetite-regulating pancreatic polypeptide receptor Y4, is present in hippocampus, and hypothalamus (Kim et al. 2014). OAS1 belongs to a part of interferon (IFN)-induced antiviral enzymes: the 2′-5′-oligoadenylate synthetases (OAS). It reported that high mRNA expression of OAS1 was tightly related to worse overall survival (OS) in grade 1 breast cancer (Zhang and Yu 2020). Several studies have indicated that a member of HOX1 family: HOXB1 play critical role in the development of cancer. It was reported that the abnormal expression of HOXB1 was associated with the onset and development of glioma (Cui et al. 2020b). Homeobox C4 (HOXC4) played an essential role in morphogenesis and differentiation during embryonic development. HOXC4 regulates EMT by regulating the transforming growth factor beta (TGF-beta) signaling in HCC (Hepatocellular carcinoma). Abnormal expression of HOXC4 has been reported in several types of cancers (Yang et al. 2021).

## Discussion

These results demonstrated that the tumor-derived exosomes can affect the biological process, cellular component and molecular function of brain organoids. These genes were enriched in cancer-related pathways, owned oncogenic signatures, and also targeted microRNA essential in the progression of cancer. It potentially implied that the tumor-derived exosomes could induce impaired neurodevelopment and might cause carcinoma of brain organoids. Metastatic transmission to placenta or fetus is most frequently seen in breast cancer, followed by bone or soft tissue sarcomas, gynecological malignancies, or other tumors (Pavlidis 2002; Alexander et al. 2003; Jackisch et al. 2003; Pentheroudakis and Pavlidis 2006; Pavlidis and Pentheroudakis 2008). Exosomes from breast cancer might be involved in the process of breast cancer metastatic transmission to placenta or fetus. We thus suppose the fetus whose mother has breast cancer during pregnancy might have the risk of neurodevelopment dysfunction after birth.

## Conclusions

In this study, we established an engineered brain organoid on chip that allowed to study the effects of cancer cells-derived exosomes on the neurodevelopment of human brain at early gestation. It revealed breast cancer derived exosomes might lead to the impairment of neurodevelopment in brain organoids, including increased stemness-related marker OCT4 of stem cell and forebrain marker PAX6 which were related to tumor progression and oncogenic features. Moreover, RNA-seq analysis revealed neurodevelopment-related signaling pathways were associated with exosomes-induced impaired neurodevelopment of brain organoids. Additionally, it reflected the activated signaling pathways associated with carcinogenesis (e.g., breast cancer and medulloblastoma). This brain organoids on chip system reveals the exosomes from breast cells might contribute to the carcinogenesis of brain organoids. Extensive work is needed to further verify these findings with in vivo models. We assume the offspring of pregnant woman with breast cancer might have increased risk of neurodevelopment dysfunction after birth.

## Methods

### Fabrication of micropillar chips

An array of micropillar chip was fabricated via conventional soft lithography procedures and micromolding according to a previous article (Cui et al. 2020a). They were designed with the top layer for sealing the chips and bottom layer composed of an array of micropillars for EBs formation. The two layers were molded by employing a PDMS pre-polymer consisting of a mixture of a curing agent (184 Silicone Elastomer, Dow Corning Corp) and PDMS monomer at the weight of 1:10 by mass. The molded pre-polymer was then polymerized via thermal curing in a dry oven for 45 min at 80 °C.

### hiPSC maintenance and culture

The hiPSCs cell line was from a human male^’^s skin fibroblasts via reprogramming, and kindly gifted from Prof. Ning Sun(Fudan University) (Cui et al. 2020a). hiPSCs were culture on Matrigel precoated (BD) six-wells plates with mTeSR1 medium (Stem cell Technologies). Cultures were passaged every 4-5 days using Accutase (Sigma). Then digested cultures were seeded on Matrigel-precoated plates and cultured with mTeSR1 medium supplemented with Y27632 (10 μM) for 1 h. Then, the culture was maintained in mTeSR1 medium.

### Generation of brain organoids

To form brain organoids, the single dissociated cells were prepared using Accutase. The cell were seeded on micropillar chips and cultures with mTeSR1 medium supplemented with 15 μM Y27632 for 2 days. At day 2, the medium was switched to KSR medium which was composed of 80% DMEM/F12 medium (Gibco), 20% KSR (Invitrogen), 0.2 mM 2-mercaptoethanol (Sigma-Aldrich), 1% minimum essential media-nonessential amino acids (MEM-NEAA, Invitrogen), 1% penicillin–streptomycin (Sigma), and 1% GlutaMAX (Invitrogen). TGF-β inhibitor A83–01(Sigma), Y27632 and Dorsomorphin (Selleck) were added into KSR medium for three days which were then replaced with 4 ng/mL bFGF (Peprotech) from day 4 to day 6. At day 6, neural induction medium (NIM) was used to replace the KSR medium. NIM was composed of DMEM/F12, 1% N2 supplement (Invitrogen),1 μg/mL heparin (Sigma), 1% GlutaMAX, 1% penicillin–streptomycin, and 1% MEM-NEAA. From day 6 to day 9, NIM were supplemented with 4 ng/mL bFGF. At day 11, the 3D cultures were cultured with neural differentiation medium.

### MCF-7 cell culture, sample preparation, and exosomes characterization

According to previous work (György et al. 2011; Chen et al. 2019), MCF-7 was maintained in medium composed of 90% DMEM (Gibco),10% exosome-depleted fetal bovine serum (FBS) (ABW, AB-FBS-ED0050), 1% GlutaMAX, 0.01 mg/mL insulin, 1% nonessential amino acid (NEAA) and 1% penicillin-streptomycin in a 5% CO_2_ humid atmosphere at 37 °C. When cells reached 80% confluent, cell medium was changed and then harvested after 48 h. Exosomes were extracted and purified according to a method described previously. Initially, to remove cell debris, the collected cell culture media from MCF-7 (about 200 mL) were centrifuged at 1000 g at 4 °C for 20 mins to obtain supernatant. To remove large macrovesicle, the supernatant was centrifuged at 10000 g for 30 mins. The resulting supernatant was filtered through a commercial 220 nm filter. Then the filtrate was centrifuged at 120000 g for 1.5 h to precipitate the exosomes at 4 °C using a SW32 Ti rotor (Beckman Coulter, USA). The resulting pellet was resuspended using 100 μLPBS and stored at −80 °C before use (Chen et al. 2019). The concentrations of exosomes were determined by nanodrop according to the manufacturer’s instructions. The particle size of purified tumor-derived exosomes was analyzed by TEM. Purified exosomes were labeled with PKH67 Cell Membrane Labeling Dye (Sigma) according to the manufacturer’s instructions and then were added into the serum-free NDM. After 24 h, the brain organoids were fixed with 4%PFA and stained with DAPI. Later, the subsequent microscopic inspection was performed.

### Treatment brain organoids with exosomes extracted from breast cancer cell

To investigate the impact of tumor-derived on the brain, the organoids randomly selected were treated with exosomes derived from breast cancer cells at the concentration of 50 μg/mL and 0 μg/mL for 5 days and 10 days respectively. Brain organoids (≥10) from one chip under different conditions were randomly selected for qRT-PCR.

### RNA extraction and qRT-PCR

To detect the gene expression level of brain-specific genes, total RNA was extracted from control organoids or exosomes-exposed organoids using RNAiso Plus (Takara). NanoDrop (Thermo Fisher Scientific) were used to measure the concentrations of RNA. PrimeScript RT Master Mix were utilized to conduct reverse transcription. qRT-PCR was conducted using Ex Taq DNA polymerase (Takara) and instrument (Bio-Rad). The primers employed in this study are demonstrated Supplementary Table 1. GAPDH serves as a reference gene.

### Tissue cryosection and immunohistochemistry

The collected brain organoids were fixed with 4% paraformaldehyde (PFA) solution overnight. At day 2, the fixed tissues were washed with PBS for 15 mins and transferred to 30% sucrose overnight. After that, they were embedded with OCT (Sakura) and then stored at −80 °C. Sections (10-15 μm thick) were cut using a cryostat (Leica) for immunohistochemistry. The sections were washed with PBS for 15 mins at room temperature to remove external OCT. For permeabilization, the washed section was soaked in 0.02% Triton X-100 diluted in PBS for 15 mins. The sections were incubated with blocking solution at 4 °C for 1 h and then were incubated with primary antibodies diluted in antibody dilutions at 4 °C for overnight. At day 2, when incubated with secondary antibodies for 1 h at room temperature, the sectioned were stained with DAPI (CST) to stain nuclei. The primary antibodies were used in this study listed as following: SOX2 (rabbit, Cell Signaling, 3579), OCT4A (Rabbit, CST, 2890S), PAX6 (rabbit, Biolegend, PRB-278P), NESTIN (mouse, Santa Cruz, sc-20978) TBR1 (rabbit, Abcam, ab31940), BRN2 (mouse, Santa Cruz, sc-393324), GABA (rabbit, Sigma, A2052, 1:400), TUJ1 (mouse, BioLegend, 801201, 1:500), vGLUT1 (mouse, Millipore, MAB5502, 1:400). Secondary antibodies Alexa Fluor 488 and 594 (anti-rabbit, anti-mouse) were used at a 1:1000 dilution. Cryosections were imaged on an Olympus FV-1000.

### Transmission electron microscopy

To examine the morphology of MCF-7 derived exosomes, the purified exosomes suspension was put on the copper grid to enable exosomes incubate with copper grid for 10 mins at room temperature. Exosomes on copper grid were rinsed with distilled water and copper grid was pipetted 10 μL uranyl acetate (2%) for 1 min. After that, filter paper was sucked off and dried under an incandescent lamp for 2 mins. Finally, we inspected and imaged the copper mesh at 80 kV using a JEM-1400 PLUS electron microscope.

### Western blot analysis

Purified exosomes were lysed in RIPA buffer system (Beyotime, P0013K) at 4 °C. Exosome’s proteins were separated by SDS-PAGE and transferred to PVDF mmembranes. The transferred membrane was incubated with rabbit anti-CD9 (Sigma-Aldrich, Cat. T6199) and rabbit anti-CD63(CST, Cat. 9516) overnight. At day 2, the HRP-linked second antibody (Cat. 7074) were used to detect the primary antibodies which were visualized using Prime Western Blotting Detection Reagent (GE life). Chemiluminescence images were acquired using ChemiDoc XRS+ System (Bio-Rad).

### Statistical analysis

Error bars indicate ±SEM. Statistical significance was determined using *t* tests, for which significance was determined with GraphPad Prism (GraphPad Software). A P value <0.05 was considered statistically significant.

## Supplementary Information

**Additional file 1: Figure S1.** Identification of MCF-7-derived exosomes with different approaches. **A,** Procedures of purification of exosomes secreted by MCF-7 using ultracentrifugation. **B,** Nanoparticle tracking analysis of exosomes produced by MCF-7 via Zetasizer Nano. **C,** Examination of exosomes specific biomarker CD9 and CD63 using western blot. **D,** Representative images of exosomes identified by transmission electron microscopy. Scale bars: 1 μm in the left image and 200 nm in the enlarged image. The arrow indicated the exosomes.

**Additional file 2: Figure S2.** RNA-seq analysis showed the oncogenic signatures in brain organoids with exosomes exposure. A, KEGG pathways of upregulated DEG genes related to cancers in brain organoids exposed to exosomes. The x-axis and y-axis represent the rich ratio and the KEGG terms, respectively. The size of the circle and color of the circle indicates the gene number and the value (adjusted *P*-value) respectively. B, Enrichment of oncogenic signatures in brain organoids exposed to exosomes using MSigDB C6. C, Enrichment of possible target of microRNA by upregulated genes in exosomes group using MSigDB C3.
**Additional file 3: Figure S3.** qRT-PCR validation of upregulated DEGs in exosomes-treated group. The mRNA expression level of NPY4R, OAS1, HOXB1, and HOXC4 were detected via qRT-PCR. Data indicate mean ± SEM. Student’s *t*-test, *P < 0.05, **P < 0.01, and ***P < 0.001.

## Data Availability

The SRA data: PRJNA724806 can be accessed via the link listed below: https://dataview.ncbi.nlm.nih.gov/object/PRJNA724806?reviewer=tn1ilvbjn19nee8d3qeg51u508. Other data and material can be acquired in the manuscript and supporting information.

## Abbreviations

hPSCs: human pluripotent stem cells
hiPSCs: human induced pluripotent stem cells
TEM: transmission electron microscope
qRT-PCR: quantitative real-time PCR
RNA-seq: RNA-sequencing
EBs: embryoid bodies
NPCs: neural progenitor cells
DEGs: differentially expressed genes
KEGG: Kyoto Encyclopedia of Genes and Genomes
IFN: interferon
OAS: oligoadenylate synthetases
OS: overall survival
HOXC4: Homeobox C4
TGF-beta: transforming growth factor beta
HCC: Hepatocellular carcinoma
