# Supplementary material for: Brain organoid-on-chip system to study the effects of breast cancer derived exosomes on the neurodevelopment of brain
Source: Cell Regen. 2022 Mar 7;11:7. doi: 10.1186/s13619-021-00102-7 (PMC8901935; doi:10.1186/s13619-021-00102-7)
Supplement: Supplementary file 1 — Additional file 1: Figure S1. Identification of MCF-7-derived exosomes with different approaches. A, Procedures of purification of exosomes secreted by MCF-7 using ultracentrifugation. B, Nanoparticle tracking analysis of exosomes produced by MCF-7 via Zetasizer Nano. C, Examination of exosomes specific biomarker CD9 and CD63 using western blot. D, Representative images of exosomes identified by transmission electron microscopy. Scale bars: 1 μm in the left image and 200 nm in the enlarged image. The arrow indicated the exosomes. [file 13619_2021_102_MOESM1_ESM.docx]

**Brain organoid-on-chip system to study the effects of breast cancer derived exosomes on the** **neurodevelopment of brain**

Kangli Cui^1, 4^, Wenwen Chen^1, 4^, Rongkai Cao^1, 4^, Yingying Xie^1, 4^, Peng Wang^1^, Yunsong Wu^1, 4^, Yaqing Wang^1^, Jianhua Qin^1, 2,3,4*^

^1^Division of Biotechnology, Dalian Institute of Chemical Physics, Chinese Academy of Sciences, Dalian, China.

^2^Institute for Stem Cell and Regeneration, Chinese Academy of Sciences, Beijing, China.

^3^CAS Center for Excellence in Brain Science and Intelligence Technology, Chinese Academy of Sciences, Shanghai, China.

^4^University of Chinese Academy of Sciences, Beijing, China.

*Correspondence should be addressed to J.Q. ([jhqin@dicp.ac.cn](mailto:jhqin@dicp.ac.cn)).

**Supplementary Information**


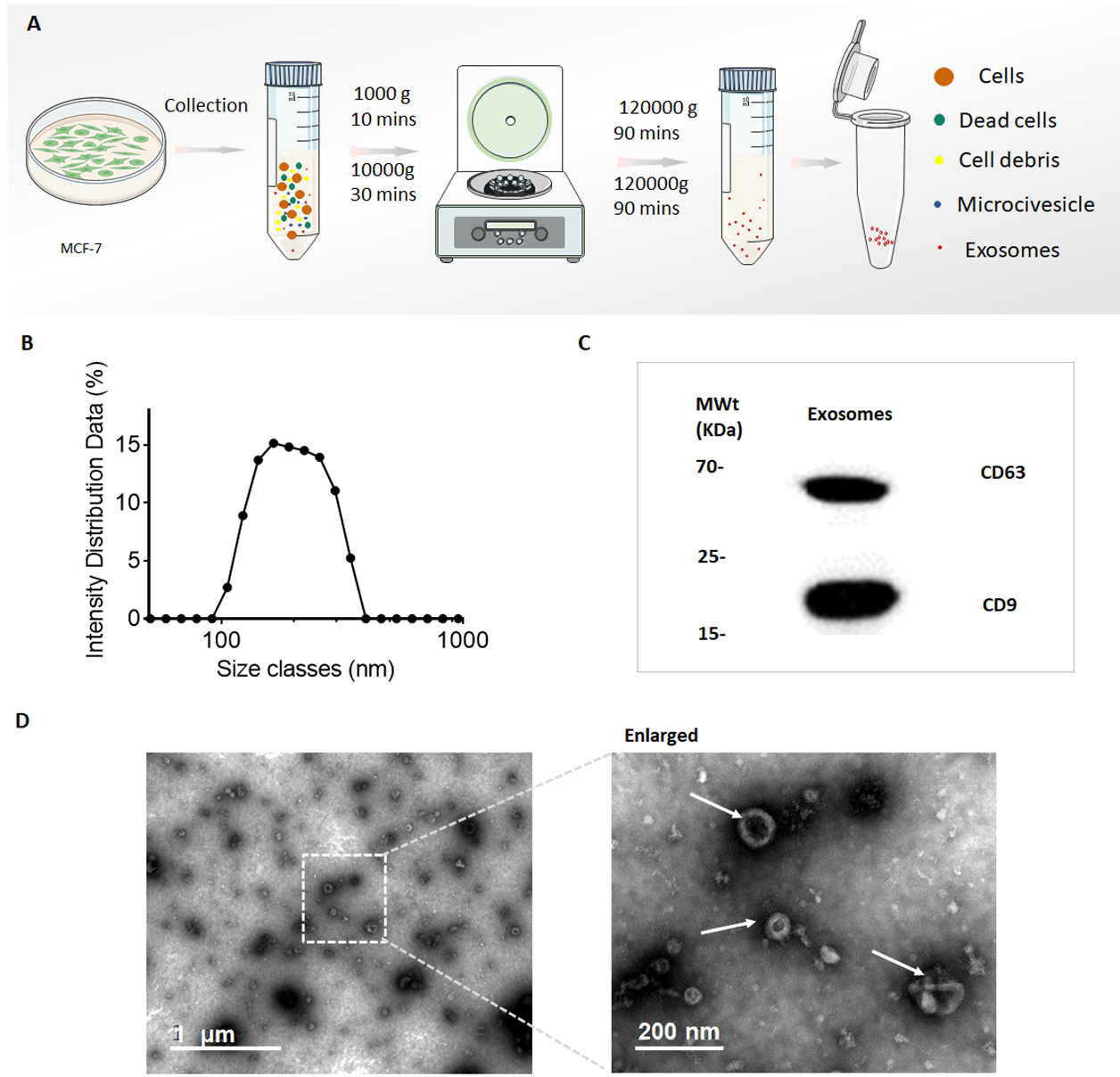


**Figure. S1** Identification of MCF-7-derived exosomes with different approaches. **A,** Procedures of purification of exosomes secreted by MCF-7 using ultracentrifugation. **B,** Nanoparticle tracking analysis of exosomes produced by MCF-7 via Zetasizer Nano. **C,** Examination of exosomes specific biomarker CD9 and CD63 using western blot. **D,** Representative images of exosomes identified by transmission electron microscopy. Scale bars: 1 μm in the left image and 200 nm in the enlarged image. The arrow indicated the exosomes.

**
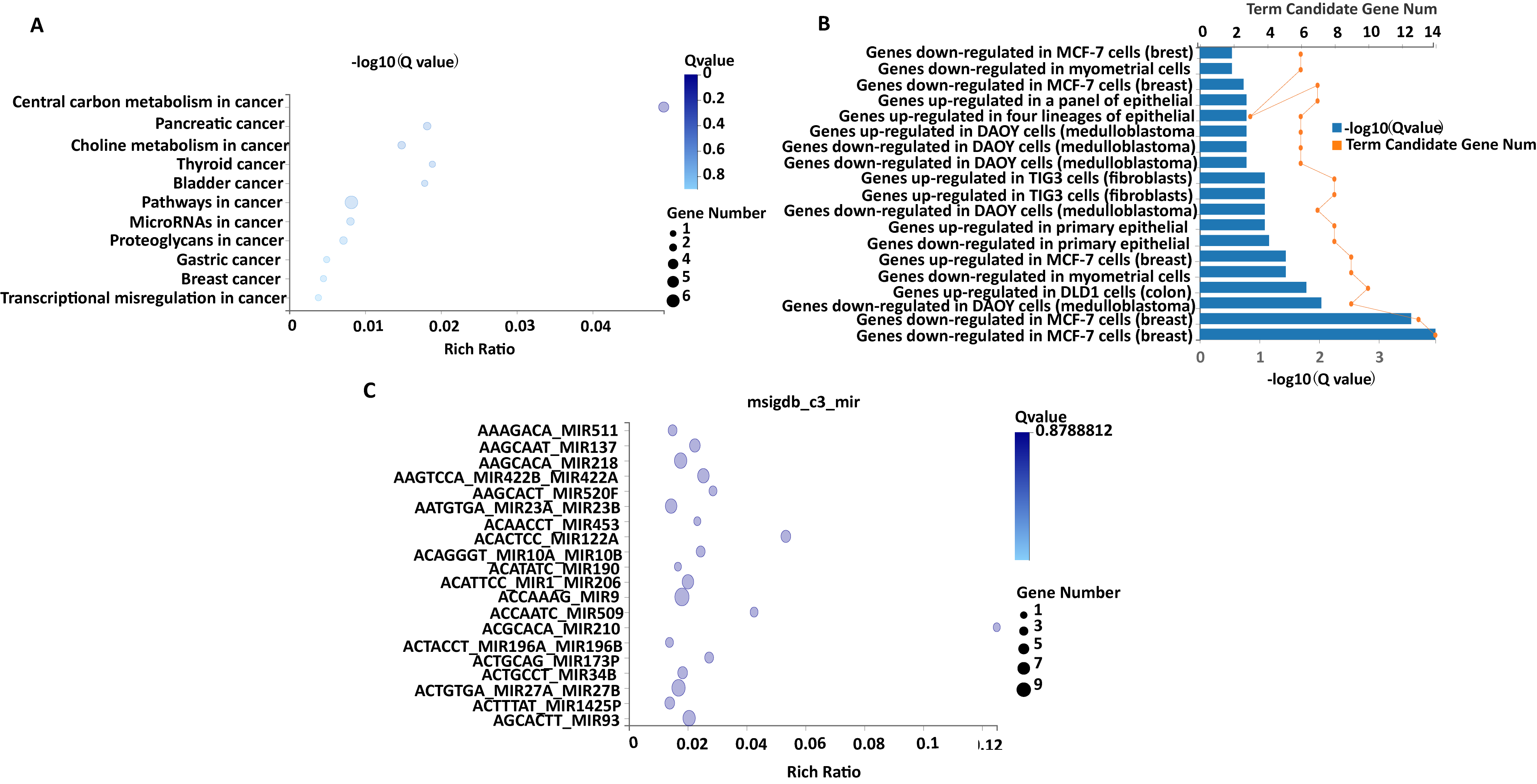
Figure S2.** RNA-seq analysis showed the oncogenic signatures in brain organoids with exosomes exposure. **A,** KEGG pathways of upregulated DEG genes related to cancers in brain organoids exposed to exosomes. The x-axis and y-axis represent the rich ratio and the KEGG terms, respectively. The size of the circle and color of the circle indicates the gene number and the value (adjusted P-value), respectively. **B,** Enrichment of oncogenic signatures in brain organoids exposed to exosomes using MSigDB C6. **C,** Enrichment of possible target of microRNA by upregulated genes in exosomes group using MSigDB C3.


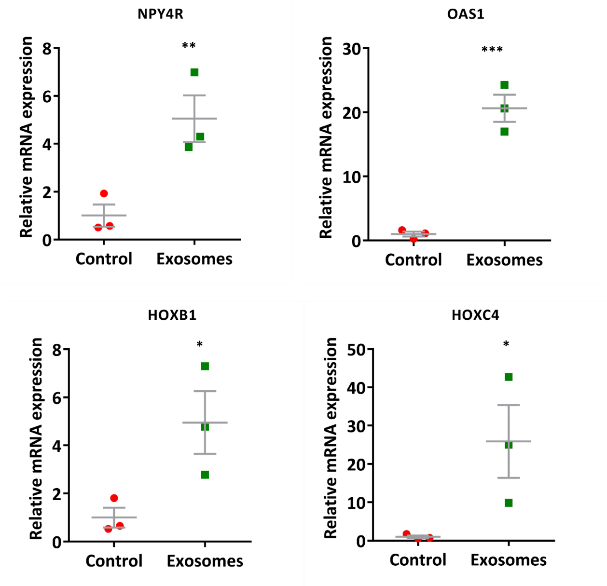


**Figure S3.** qRT-PCR validation of upregulated DEGs in exosomes-treated group. The mRNA expression level of NPY4R, OAS1, HOXB1, and HOXC4 were detected via qRT-PCR. Data indicate mean ± SEM. Student’s *t*-test, *P< 0.05, **P< 0.01, and ***P< 0.001.
